# Determination of the energy expenditure, sources, and loss of water among young adults

**DOI:** 10.1186/s12986-022-00668-2

**Published:** 2022-05-02

**Authors:** Na Zhang, Hairong He, Jianfen Zhang, Guansheng Ma

**Affiliations:** 1 Department of Nutrition and Food Hygiene, School of Public Health, Peking University, 38 Xue Yuan Road, Hai Dian District, Beijing, 100191 China; 2 Laboratory of Toxicological Research and Risk Assessment for Food Safety, Peking University, 38 Xue Yuan Road, Hai Dian District, Beijing, 100191 China

**Keywords:** Energy expenditure, Water, Water source, Water loss, Doubly labeled water

## Abstract

**Background:**

Few studies on measuring human energy expenditure with the doubly labeled water method has been conducted in China. The sources and loss of water among human body have never been systematically evaluated. Less data can be available for the development of the recommendation on energy expenditure and water intake. The objective of this study was to determine the energy expenditure, water sources, and loss among young adults.

**Methods:**

In this cross-sectional study, 25 participants were recruited. Double-labeled water was used to determine their energy expenditure. Water loss through skin evaporation and respiration of the lungs and water sources from metabolic water were calculated using corresponding formula, respectively. Water loss through excretion of urine was recorded and evaluated using “3-day, 24-h, real-time urine excretion record” method. All urine samples were collected and weighed in the 3 days. Water loss through excretion of feces was evaluated using “3-day, 24-h, real-time fecal-excretion record” method. All fecal samples were collected and tested by the direct drying method. Water sources from fluid intake were recorded by “7-day, 24-h, real-time fluid intake record” method. Water intake from food was calculated and tested by the weighing method combined with the duplicate portion method and the direct drying method in the 3 days.

**Results:**

The energy expenditure of males was 2187 kcal/d, and that of females was 1987 kcal/d. The median fluid intake, water intake from food, and metabolic water were 887, 1173 and 246 mL, respectively, which accounted for 38.8%, 50.3%, and 11.2% of total water sources. There was a gender difference in the percentage of metabolic water (*Z* = − 2.135, *P* = 0.033). The water loss through urine excretion, skin evaporation, respiration, and feces excretion was 1295, 172, 149 and 64 mL, respectively, which accounted for 76.5%, 10.3%, 9.5%, and 3.6% of the total water losses. Gender differences in the amount of water loss through respiration and skin evaporation were found (*Z* = − 4.125, *P* < 0.001; *Z* = − 3.723, *P* < 0.001).

**Conclusions:**

Energy expenditure of male was higher than that of female. The first major water sources was water intake from food in this study, and the first major water loss was urine excretion.

*Trial registration* The study was registered on the website of Chinese clinical trial registry, and the code of identification is ChiCTR1900028746.

**Supplementary Information:**

The online version contains supplementary material available at 10.1186/s12986-022-00668-2.

## Introduction

Water, as an easily neglected nutrient, is an indispensable nutrient for human survival and development [1]. Water has a variety of physiological functions in the human body, including participating in the process of metabolism, regulating the osmotic pressure, maintaining normal body temperature, and so on [1]. Water balance refers to the dynamic equilibrium state in which the water intake and water loss are roughly equal, which is vital to keep health [1]. Both dehydration and excessive water can affect health [1]. Dehydration reduces the body's cognitive ability and physical activity and increases the risk of urinary and cardiovascular diseases [2]. Although the phenomenon of excessive water is rare, serious cases endanger life and health [1]. Therefore, the formulation of recommendations on water intake is very important to guide residents to drink enough water and keep healthy. There are three main methods to develop recommendations on adequate water intake [1]. The first method is to calculate water requirements according to the relationship between energy expenditure and water metabolism. The second method is to calculate water requirement according to the amount of water loss. There are few data on water loss. It is necessary to adopt scientific methods to obtain reference data. The third method is to develop the recommendation based on the related water intake data. The determination of energy expenditure and the source and loss of water can provide reference data for the development of the recommendation on adequate water intake.

Since the doubly labeled water method was successfully applied to animal experiments in the 1950s, this has been recognized by researchers at home and abroad as the "gold standard" for measuring people's total energy expenditure (TEE) under free living conditions and is often used to verify the accuracy of other methods [3, 4]. However, there are few studies on measuring human energy expenditure with the doubly labeled water method in China, and more scientific and accurate reference data need to be accumulated. About the determination of water intake and water loss, there are three sources of water for the human body, including fluid intake, water intake from food, and metabolic water. The method of “7-day, 24-h, real-time fluid intake record” (Liq.In7) is applicable to measure fluid intake for individuals [5]. Several studies have verified the accuracy and effectiveness of this method [6–8]. The most accurate measurement method of water intake from food was the combined weighing method and duplicate portion method [5, 9]. Metabolic water is mainly produced by the metabolism of three energy-supplying substances-carbohydrates, fats, and proteins-in the human body, so it is closely related to TEE and dietary structure [10]. At present, there is no method to directly determine the amount of metabolic water in the human body, which can only be calculated by corresponding formulas with accurate determination on the TEE as coefficient [11]. Water in the human body is lost in four ways: urine excretion, skin evaporation, respiration, and feces excretion. Water loss of urine excretion can be collected in real-time by participants. The skin mainly excretes water from the body in the form of sweating [12]. The forms of skin evaporation can be divided into dominant sweating and non-dominant sweating. Dominant sweating refers to the sweat secretion of human sweat glands due to reasons, such as being in a high-temperature environment and mental excitement. The amount of dominant sweating is related to factors such as physical activity intensity, ambient temperature, and humidity [13]. Non-dominant sweating refers to the loss of water evaporated through the skin surface when there is no activity of sweat glands [14]. At present, there have been explorations on the measurement methods of non-dominant sweating at home and abroad, such as the method of a dynamic box and the method of a condenser [15]. Some formulas can also be used to determine the amount of water loss through skin evaporation using absolute humidity and body surface area as coefficients [11, 16]. Water loss through respiration can be calculated using a corresponding formula with respiratory volume and absolute humidity as coefficients [11, 12, 16]. There were little data on the determination of fecal water content at home and abroad. The main factors affecting the amount of water loss through the digestive tract are the amount and water content volume of feces. Generally, people defecate once a day, about 100–300 g, which is affected by the3 type and amount of food intake [17, 18]. Theoretically, it can be evaluated that water loss through feces excretion is 25–200 g among people with a normal gastrointestinal function every day. Water loss from feces excretion can be determined more accurately using the direct drying method.

In this study, the primary objective was to determine the TEE of young adults using the doubly labeled water method. The secondary objective was to measure the water sources and losses of the human body. These data could provide useful reference data for the development of recommendations on energy value and water intake.

## Materials and methods

### Sample size calculation

The sample size was calculated using the formula $\mathrm{N}={\left[\frac{{Z}_{\alpha /2}CV}{\varepsilon }\right]}^{2}$. For the formula, α was set as 0.05$, \varepsilon$ was the maximum relative error and was set as 10%$,$ and *CV was the coefficient of variation* and was set as 0.15 according to the previous literature [19]. Considering the possible missed follow-up rate of 10%, finally, a total of 20 participants was needed.

### Participants

A total of 25 young adults aged 18–23 years were recruited by offline campus propaganda from one college in Baoding, Hebei Province, China.

The inclusion criteria were as follows: the age of participants was between 18 and 23 years; the participants were in a healthy state. The exclusion criteria were as follows: the age of participants were < 18 years or > 23 years; the participants had a history of smoking and drinking alcohol or performing intensive physical activities (> 6 METs); the participants had diseases of the gastrointestinal tract or of the kidney, cognitive disorders, or other chronic and metabolic diseases. Females who were being in menstrual cycle or pregnant were also excluded.

### Ethics

The study protocol and instruments were reviewed and approved by the Ethical Review Committee of Peking University. The code of identification is IRB00001052-19158. The study was registered on the website of Chinese clinical trial registry, and the code of identification is ChiCTR1900028746. The study was conducted in accordance with the guidelines of the Declaration of Helsinki. Prior to the conduction of the study, all the participants read and voluntarily signed informed consent.

### Study design and procedure

**Fig. 1:**
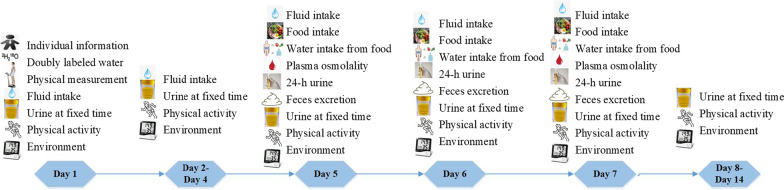
The flow diagram

The cross-sectional study lasted 14 days. At 6 a.m. on the first day, the morning urine samples of the participants were collected and tested as baseline data. The height and weight of the participants were measured to calculate their body mass index (BMI). According to the dose of 1 g/kg·bw (body weight), the double-labeled water (^2^H 7.96 atom%/^18^O 9.81 atom%, Jiangsu Huayi Technology, Xiamen, China) was weighed by one ten-thousandth analytical balance (ES-E, Shanghai, China), and was drunk by the participants at 7 a.m. for isotope labeling. Then, 2 mL urine sample was collected 2 h, 4 h, 6 h, and 8 h after taking double-labeled water. Over the next 13 days, 2 mL of urine was collected at 7:30 a.m. every morning from day 2 to 14 to test isotopic abundance. The “7-day, 24-h, real-time fluid intake record “ (liq. In7) was used from day 1 to day 7, and the water-intake behaviors of the participants were evaluated and recorded using uniformly customized cup with scale to the nearest of 5 mL as reference. All the participants' feelings of thirst were evaluated by the visual analog score questionnaire when water-intake behaviors happened. From day 5 to day 7, the weighing method combined with duplicate proportion method was used to weigh and record all foods taken by the participants, and the direct drying method was used to measure the water intake from food. The corresponding chemical analysis method was used to determine the protein, fat, carbohydrate, and total ash in the food, respectively. The "3-day, 24-h, real-time urine-excretion record" and "3-day, 24-h, real-time fecal-excretion record" were used to record all the urine and feces excretion samples among the participants in real-time for 3 days. All urine and feces excretion samples were weighed. The direct drying method was used to measure the water content in feces. Cubital venous blood was collected on day 5, and plasma osmolality was determined. From day 1 to day 14, the physical activity record form was filled in to analyze the physical activity among the participants. In addition, data on the temperature, humidity, and wind speed of the environment where the participants located during these days were recorded in real-time. In the whole process of the study, participants who failed to meet these requirements needed to let investigators know. Finally, all participants finished the study, and no one failed to meet the requirements. The indicators collected at different time points in the study are shown in Table 1. The flow diagram was shown in Fig. 1.

**Table 1:** The indicators collected at different time points in the study

	Day 1	Day 2	Day 3	Day 4	Day 5	Day 6	Day 7	Day 8	Day 9	Day 10	Day 11	Day 12	Day 13	Day 14
Individual information	√													
Doubly labeled water	√													
Physical measurement	√													
Fluid intake	√	√	√	√	√	√	√							
Food intake					√	√	√							
Water intake from food					√	√	√							
Plasma osmolality					√		√							
24 h urine					√	√	√							
Feces excretion					√	√	√							
Urine at fixed time	√	√	√	√	√	√	√	√	√	√	√	√	√	√
Physical activity	√	√	√	√	√	√	√	√	√	√	√	√	√	√
Environment	√	√	√	√	√	√	√	√	√	√	√	√	√	√

### Anthropometric measurements

Weight and height were measured by trained investigators according to relevant standards and procedures. Wearing light clothing and no footwear, height was measured twice with 0.1 cm accuracy, and weight was measured twice with 0.1 kg accuracy using a height–weight meter (HDM-300; Huaju, Yiwu, Zhejiang, China).

$${\text{Body}}\;{\text{Mass}}\;{\text{Index}}\;({\text{BMI}},\;{\text{kg}}/{\text{m}}^{{2}} ) = {\text{weight}}\;\left( {{\text{kg}}} \right)/{\text{height}}\;{\text{squared}}\;\left( {{\text{m}}^{{2}} } \right).$$

### Assessment of fluid intake

The method of “7-day, 24-h, real-time fluid intake record” (Liq.In7) has been verified to be accurate and effective for evaluating fluid intake and used in this study [20, 21]. The participants were trained to record all fluid intake behaviors for 7 consecutive days. The type, amount, time, and place of fluid intake were recorded in detail. For assisting the participants to evaluate the amount of fluid intake, uniformly customized cups with scale to the nearest of 5 mL were provided for every participant as reference. All types of fluid, including tap water, bottled water, and other beverages, were counted.

### Assessment of dietary intake

All food in 3 consecutive days, including two working days and one non-working day, was recorded and weighted using electronic balance (YP20001, SPC, Shanghai, China). The weight of each food before and after eating (including inedible and inedible parts, such as bones) was weighted, and then the actual amount was calculated (g). Using the method of duplicate portion sampling, the same backup food samples were collected for testing of macronutrient intake and water intake from foods [22].

Test of water intake from foods: The water content in food was tested using the direct drying method according to the *National Food Safety Standard GB 5009.3-2016 Determination of W*ater in Foods [23]. Parallel samples were tested for each kind of food, and the error of two test results did not exceed 5%. Then, the percentage of water content in food and water intake from food were calculated [22].

$$\begin{aligned} & The\;percentage\;of\;water\;content\;in\;food\;\% \\ & \quad = \left( {weight\;of\;food\;sample\;before\;drying} \right. \\ & \left. {\quad \quad {-} \, weight\;of\;food\;sample\;after\;drying} \right)/weight\;of\;food\;sample\;before\;drying \times 100\% . \\ \end{aligned}$$

$$\begin{aligned} & Water\;{\text{int}} ake\;from\;food\;\left( {{\text{mL}}} \right) \\ & \quad = the\;actual\;amount\;of\;food\;{\text{int}} ake \\ & \quad \quad \times \, the\;percentage\;of\;water\;content\;in\;food\;\% \div 1.0\;{\text{g/mL}}. \\ \end{aligned}$$

Test of energy value from food: The protein content in food was tested using the Kjeldahl determination method according to the *National Food Safety Standard GB 5009.5-2016 Determination of protein in Food* [24]. The fat content in food was tested using the acid hydrolysis method according to the *National Food Safety Standard GB 5009.6-2016 Determination of fat in Food* [25]. The ash content in food was tested using the method according to the *National Food Safety Standard GB 5009.4-2016 Determination of ash in Food* [26]. The carbohydrate content in food was calculated by subtracting the total weight of the food sample from the weight of protein, fat, ash, and water in the food sample. Parallel samples were determined for each type of food, and the error of two test results did not exceed 5%.

$$\begin{aligned} Energy\;value\;from\;food\;\left( {\text{kJ/d}} \right) & = Intake\;of\;protein\;\left( {\text{g/d}} \right) \times 4 \times 4.184 \\ & \quad + Intake\;of\;fat\;\left( {\text{g/d}} \right) \times 9 \times 4.184 \\ & \quad + Intake\;of\;carbohydrate\;\left( {\text{g/d}} \right) \times 4 \times 4.184 \\ \end{aligned}$$

### Determination on urine samples

Collection of urine samples: All urine samples were collected in disposable urine storage bags by participants in 3 days, and then the samples were stored at + 4 °C [22].

Measurement of water loss from urine excretion: Starting with the second voiding on one day and ending with the first voiding on next day, all urine samples were collected as total 24 h urine volume, which was calculated as water loss through urine excretion in a day. Urine volume was measured with the accuracy of 0.1 kg with electronic desktop scale (YP20001; SPC; Shanghai, China).

### Determination on plasma osmolality

Cubital venous blood was also used to test plasma osmolality, which can reflect the hydration status. When the plasma osmolality is less than or equal to 290 mOsm/kg, the body is in a normal hydration state. When the plasma osmolality is > 290 mOsm/kg, the body is in a dehydration state [2]. Plasma osmolality was determined using osmolality meter (SMC 30C, Tianhe, Tianjin, China).

### Determination on feces samples

Collection of feces samples: All feces samples were collected in disposable black fecal collection box by participants for 3 days, and then the samples were stored at + 4 °C.

Measurement of water loss from feces excretion: Feces samples were measured with an accuracy of 0.1 kg with electronic desktop scale (YP20001; SPC; Shanghai, China). Additionally, the water content in feces was tested using the direct drying method according to the *National Food Safety Standard GB 5009.3–2016 Determination of Water in Foods* [23]. The difference between the original weight of fecal sample and the constant weight after drying was the water content of fecal sample.

### Determinations on isotopic abundance

A stable isotope mass spectrometer (MAT253 plus; Thermo Fisher Scientific; Massachusetts, USA) and multi-purpose on-line gas preparation and introduction device (Gas BenchII; Thermo Fisher Scientific; Massachusetts, USA) were used to determine the isotopic abundance in the urine of the participants [19, 27].

Analysis of hydrogen isotopes using H_2_-H_2_O equilibrium method: The chromatographic column temperature was set at 70 °C. The procedure was as follows: 0.2 mL urine sample was transferred to a 12 mL sample bottle using a pipette, then hydrophobic platinum catalyst was added in, the bottle cap was tightened, and 2% H_2_ and He mixture was filled in for 600 s. After inflation, the bottle stood at 25 °C for 1 h. Then, after the isotopic exchange between gas H_2_ and hydrogen, gas H_2_ and miscellaneous gas were separated through the chromatographic column before entering the stable isotope mass spectrometer for testing. Three national reference samples, including QYTB (Beijing water), QYTB1 (seawater), and QYTB2 (Tibet water), were used in the test. The accuracy of hydrogen isotope analysis of standard samples reached 0.2‰.

Analysis of oxygen isotopes by CO_2_-H_2_O equilibrium method: The chromatographic column temperature was set at 70 °C. The procedure was as follows: 0.3% CO_2_ and He mixture was filled in the 12 mL sample bottle for 600 s. After inflation, 0.2 mL of sample was injected into the 12 mL sample bottle filled with balance gas and stood for 12 h at 25 °C. After the isotopic exchange between oxygen in gas CO_2_ and oxygen in samples, gas CO_2_ and miscellaneous gas were separated through the chromatographic column before entering the stable isotope mass spectrometer for testing. Three national reference samples, including QYTB (Beijing water), QYTB1 (seawater), and QYTB2 (Tibet water), were used in the test. The accuracy of oxygen isotope analysis of standard samples reached 0.2‰.

### Evaluation of other influencing factors

*The determination on subjective thirst sensation and urinary urgency, physical activity,* temperature, humidity, and wind speed is described in the Additional file 1.

### Quality control

Unification of study procedure and training: Standardized training was conducted for investigators to make them familiar with all procedures of each step. Unified training was conducted for the participants to make them understand how to fill in the corresponding questionnaires, how to collect relevant samples, and be familiar with the procedures that they need to participate in. After the study began, the whole process was strictly supervised and quality controlled by special quality-control researchers.

Quality control on the procedure of double-labeled water: The error of the double-labeled water concentration used in this study was accurate to within ± 0.5%, and the production process met the standards and requirements of Current Good Manufacture Practices (cGMP). At 6:00 a.m. on day 1, the participants were required to measure their weight after emptying morning urine. The amount of double-labeled water for each participant was calculated accurately according to the weight. At the same time, 100 mL of distilled water for mouthwash was weighed for each participant. The participants drank and swallowed the double-labeled water within the specified time and rinsed their mouths according to the specified process. The method of determination on isotopic abundance was accurate. During the test, when the internal accuracy was lower than 0.1%, the retest was started. The external accuracy of the data and the stability of the instrument were tested for the parallel samples every 10 samples.

### Statistical analysis

SAS 9.2 (SAS Institute Inc., Cary, NC, USA) was used. The mean and standard deviation (SD) were used to describe the quantitative parameters in line with the normal distribution, and a T-test was used to analyze the indexes of different groups of participants. The median and interquartile were used to describe the quantitative parameters not in line with the normal distribution, and Mann–Whitney U test was used to analyze these indexes of different groups of participants. Count data were presented as n (percentage), and the method of Chi-square test was used to analyze these indexes of different groups of participants. The significance levels were set at 0.05. The formulas on the indexes related to the determination of doubly labeled water, the calculation on the metabolic water, water loss through skin evaporation, and respiration are described in the Additional file 2.

## Results

### Participants characteristics and the environment

All participants finished the study. The average age of these 25 young adults was 20.9 ± 1.1 years. The height, weight, BMI, body surface area, and physical activity were 165.2 ± 8.9 cm, 60.1 ± 8.4 kg, 22.1 ± 2.8 kg/m^2^, 1.65 ± 0.14 m^2^, and 2067 ± 554 MET-min/w (Table 2). There was statistical significance in height, weight, body surface area, and physical activity when comparing between males and females (*t* = − 5.042, *P* < 0.001; *t* = − 3.345, *P* = 0.003; *t* = − 5.172, *P* < 0.001; *t* = − 2.466, *P* = 0.022).

**Table 2:** Characteristics of participants

	Age (y)	Height (cm)	Weight (kg)	BMI (kg/m^2^)	Body surface area (m^2^)	Physical activity (MET-min/w)
Total (n = 25)	20.9 ± 1.1	165.2 ± 8.9	60.1 ± 8.4	22.1 ± 2.8	1.65 ± 0.14	2067 ± 554
Female (n = 14)	21.1 ± 1.2	159.6 ± 6.5	56.0 ± 6.3	22.0 ± 2.7	1.57 ± 0.10	1847 ± 587
Male (n = 11)	20.6 ± 1.0	172.4 ± 6.0	65.4 ± 7.8	22.1 ± 3.2	1.77 ± 0.10	2348 ± 366
*t*	1.052	− 5.042	− 3.345	− 0.060	− 5.172	− 2.466
*P*	0.297	< 0.001*	0.003*	0.953	< 0.001*	0.022*

Values are shown as the mean ± standard deviation (SD)

*Statistically significant differences, *P* < 0.05

The average temperature indoors and outdoors of the dormitory and classroom, canteen, and playground from day 1 to 14 was 20.7 °C, 20.3 °C, 21.1 °C, 20.9 °C, 21.4 °C and 18.2 °C, respectively. The average humidity indoors and outdoors of the dormitory and classroom, canteen, and playground was 65.2% RH, 44.7% RH, 50.7% RH, 48.2% RH, 51.8% RH, and 42.7% RH, respectively. The average wind speed of the dormitory, classroom, canteen, and playground was 0.0 m/s, 0.0 m/s, 0.0 m/s, and 1.0 m/s, respectively. According to the corresponding table of absolute humidity and relative humidity (atmospheric pressure: 1 bar), the absolute humidity was calculated as 8.64 g/m^3^ (20 °C, 50% RH).

### Behaviors of water intake and urine excretion

Among 25 participants, the median number of 24 h fluid intake was 5 (2). The percentage of no sensation of thirst, initial sensation of thirst, a strong sensation of thirst, and super sensation of thirst was 5.1%, 73.3%, 19.2%, and 0.0%, respectively, and statistical significance was found between genders (χ^2^ = 32.940, *P* < 0.001). The median 24 h urination was 6 (2). The percentage of no sensation to urinate, initial sensation or urge to urinate, strong urge to urinate, and super urge to urinate was 6.2%, 43.2%, 46.3%, and 0.0%, respectively (Table 3). The average plasma osmolality was 290.1 ± 2.2 mOsm/kg among 25 participants. No statistically significant difference was found in plasma osmolality between females and males (289.6 ± 2.4 *VS* 290.7 ± 1.8, mOsm/kg, *t* = 1.268, *P* = 0.217) (Table 3).

**Table 3:** Behaviors of water intake and urine excretion among participants

	Number of fluid intakes/24 h	Thirst sensation % M (QR)				Number of urinations/24 h	Urinary urgency % M (QR)			
	M (QR)	1	2	3	4	M (QR)	1	2	3	4
Total (n = 25)	5 (2)	5.1 (12.2)	73.3 (21.2)	19.2 (17.8)	0.0 (0.0)	6 (2)	6.2 (9.8)	43.2 (32.1)	46.3 (30.4)	0.0 (2.1)
Female (n = 14)	5 (3)	11.2 (9.1)	68.9 (16.9)	13.8 (14.7)	0.0 (0.0)	6 (3)	6.1 (9.7)	43.9 (26.2)	47.9 (30.1)	0.0 (7.2)
Male (n = 11)	5 (3)	0.0 (4.8)	79.1 (43.3)	21.2 (28.4)	0.0 (0.0)	5 (2)	6.2 (10.0)	37.2 (44.1)	45.8 (39.6)	0.0 (0.0)
*Z/*χ^2^	0.192^a^	32.940^b^				1.485^a^	5.975^b^			
*P*	0.848	< 0.001*				0.149	0.118			

*Statistically significant differences, *P* < 0.05

^a^Statistic value of Mann–Whitney U test

^b^Statistic value of χ^2^ test

### Energy value and TEE

Among 25 participants, the median protein, fat, and carbohydrate intake was 51.2 (10.0), 63.0 (17.9), and 250.0 (53.5) g/d, respectively. The energy provided by protein, fat, and carbohydrate accounted for 11.3%, 33.0%, and 55.9% of the total energy value, respectively. The median energy value and TEE were 7134.3 (1766.4) and 7800.2 (1911.2) kJ/d, respectively. There was a statistical difference in the energy value and TEE between females and males (6735.7 kJ *VS* 7563.5 kJ, *Z* = − 2.682, *P* = 0.007; 7362.3 kJ *VS* 8275.5 kJ, *Z* = − 2.682, *P* = 0.007) (Table 4).

**Table 4:** Food intake and TEE of participants

	Protein		Fat		Carbohydrate		Energy value	VCO_2_	VO_2_	TEE
	g/d	Energy%	g/d	Energy%	g/d	Energy%	kJ/d	L/g	L/g	kJ/d
Total (n = 25)	51.2 (10.0)	11.3 (1.6)	63.0 (17.9)	33.0 (6.9)	250.0 (53.5)	55.9 (6.3)	7134.3 (1766.4)	332.4 (77.5)	384.3 (95.7)	7800.2 (1911.2)
Female (n = 14)	49.5 (8.4)	11.8 (2.0)	58.6 (20.4)	34.9 (7.4)	231.9 (62.7)	53.5 (7.5)	6735.7 (1502.4)	319.8 (71.8)	360.6 (71.8)	7362.3 (1646.0)
Male (n = 11)	56.8 (10.0)	10.9 (1.5)	66.4 (12.7)	31.8 (5.3)	277.9 (79.3)	57.2 (5.0)	7563.5 (1758.5)	359.5 (88.2)	405.7 (93.9)	8275.5 (1939.4)
*Z*	− 1.972	− 1.588	1.177	− 1.588	− 3.011	− 1.642	− 2.682	− 2.737	− 2.682	− 2.682
*P*	0.049*	0.112	0.239	0.112	0.003*	0.101	0.007*	0.006*	0.007*	0.007*

*Statistically significant differences, *P* < 0.05

### Water sources among participants

**Fig. 2:**
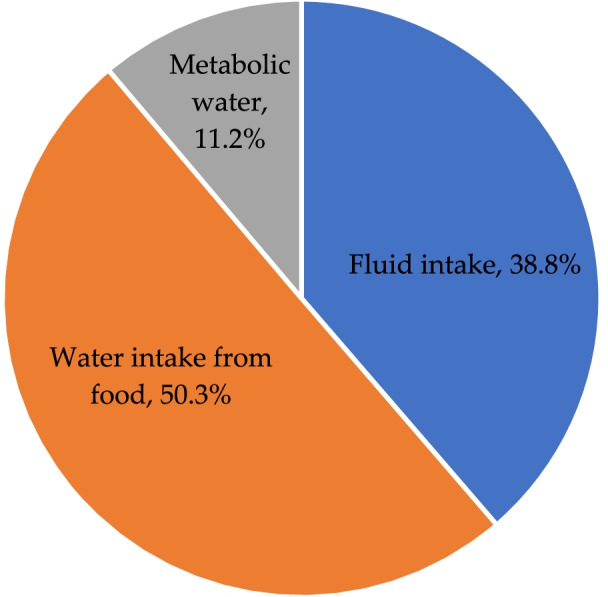
Pie chart of percentage of water sources in different ways

Among 25 participants, the median fluid intake, water intake from food, and metabolic water were 887 (538), 1173 (219), and 246 (4) mL/d, respectively. The amount of fluid intake, water intake from food, and metabolic water accounted for 38.8%, 50.3%, and 11.2% of the total water sources, respectively. There was a statistical difference in the percentage of metabolic water and TEE between females and males (11.8% *VS* 10.6%, *Z* = − 2.135, *P* = 0.033; 2059 mL *VS* 2377 mL, *Z* = − 2.135, *P* = 0.033) (Table 5 and Fig. 2).

**Table 5:** Water sources among participants

	Fluid intake		Water intake from food		Metabolic water		The total water sources
	Amount (mL/d)	Percentage (%)	Amount (mL/d)	Percentage (%)	Amount (mL/d)	Percentage (%)	Amount (mL/d)
Total	887 (538)	38.8 (16.6)	1173 (219)	50.3 (16.5)	246 (4)	11.2 (3.0)	2221 (712)
Female	767 (450)	38.2 (18.1)	1127 (231)	50.9 (19.7)	244 (4)	11.8 (2.8)	2059 (541)
Male	979 (568)	40.3 (13.5)	1225 (314)	50.3 (11.0)	244 (3)	10.6 (2.4)	2377 (569)
*Z*	− 1.533	− 0.712	− 1.588	− 0.438	− 1.752	− 2.135	− 2.135
*P*	0.125	0.477	0.112	0.661	0.080	0.033*	0.033*

Values are shown as the median and interquartile range (Q3–Q1)

*Statistically significant differences, *P* < 0.05

### Water losses among participants

**Fig. 3:**
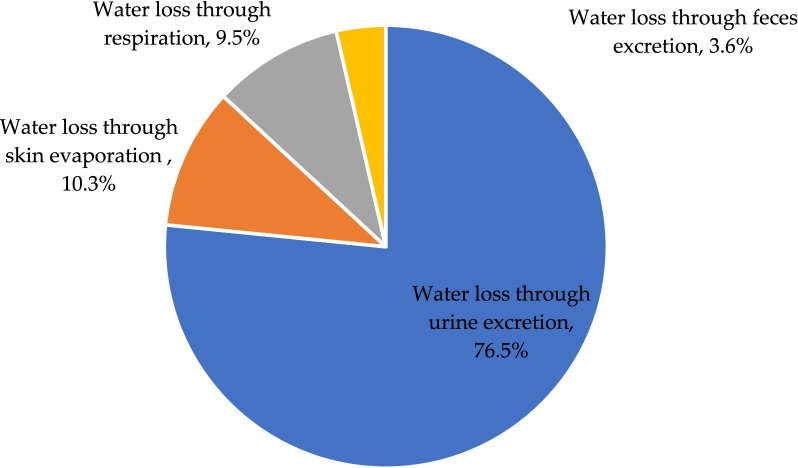
Pie chart of percentage of water losses in different ways

Among 25 participants, the median water loss through urine excretion, skin evaporation, respiration, and feces excretion was 1295 (569), 172 (24), 149 (31), and 64 (84) mL/d, respectively. The amount of water loss through urine excretion, skin evaporation, respiration, and feces excretion accounted for 76.5%, 10.3%, 9.5%, and 3.6% of the total water loss, respectively. There was a statistical difference in the amount of water loss through skin evaporation and respiration and the total water loss between females and males (160 mL VS 181 mL, Z = − 3.723, *P* < 0.001; 145 mL VS 174 mL, Z = − 4.125, *P* < 0.001) (Table 6 and Fig. 3).

**Table 6:** Water losses among participants

	Water loss through urine excretion		Water loss through skin evaporation		Water loss through respiration		Water loss through feces excretion		The total water losses
	Amount (mL)	Percentage (%)	Amount (mL)	Percentage (%)	Amount (mL)	Percentage (%)	Amount (mL)	Percentage (%)	Amount (mL)
Total	1295 (569)	76.5 (8.6)	172 (24)	10.3 (3.3)	149 (31)	9.5 (3.6)	64 (84)	3.6 (4.1)	1752 (530)
Female	1346 (597)	78.3 (7.8)	160 (18)	9.3 (3.8)	145 (6)	8.5 (3.5)	64 (46)	3.4 (3.2)	1718 (654)
Male	1255 (544)	73.8 (11.6)	181 (8)	10.8 (2.4)	174 (7)	10.1 (2.7)	84 (105)	6.1 (6.6)	1752 (447)
*Z*	− 0.328	− 1.314	− 3.723	− 0.712	− 4.125	− 1.040	− 0.821	− 0.876	− 0.602
*P*	0.767	0.202	< 0.001*	0.501	< 0.001*	0.291	0.434	0.403	0.572

Values are shown as the median and interquartile range (Q3–Q1)

*Statistically significant differences, *P* < 0.05

## Discussion

In this study, the energy expenditure, sources, and loss of water among young adults were determined**.** This study showed that the median TEE of boys and girls was 2187 kcal/d and 1987 kcal/d, respectively. In China, the data of direct determination of total energy expenditure by doubly labeled water method are limited. The TEE of females in this study was higher than that of 16 young women (1827 kcal/d) reported in a study conducted in 2010 [19]. The result of another study in 2013 using the method of doubly labeled water study suggested that the TEE of 16 young men was 2258 ± 180 kcal/d [28]. Some studies have also pointed out that although the results of the doubly labeled water method were more accurate, the results were also affected by region, physical activity, food intake, physical health status, and so on. The TEE of adults in South and North China was measured by the doubly labeled water method, and the mean values were 2052 kcal/d and 1893 kcal/d, respectively [29]. The increase in physical activity among people also significantly increases their TEE. According to the survey carried out among wrestlers in a Japanese university, the average TEE was as high as 4283 kcal/d, which was similar to that in a study conducted among football players (4369 kcal/d) [11, 30]. The research conducted by Luo Wei et al. showed that the average TEE of inpatient rehabilitation personnel (154 kJ/kg/d) was lower than that of normal personnel (192–209 kJ/kg/d) [31]. In 1989, it was proposed by the United States that water requirement was 1–1.5 mL/4.184 kJ according to the energy expenditure. Thus, water requirement of participants in this study can be calculated.

The results of this study showed that the proportion of metabolic water accounting for the total water sources was the smallest, which was only about one-tenth of the total water sources. This study found that the total water intake of young adults was 2221 mL, of which the median daily fluid intake was 887 mL, and the median water intake from food was 1173 mL. Compared with the results of studies carried out in similar populations in the same region, the average daily fluid intake was 1342 mL, and the average water intake from food was 1211 mL among young male adults in Hebei Province in a study conducted in 2016 [9]. The results of another study in 2018 found that the daily fluid intake was 1135 mL and the water intake from food was 1173 mL [32]. Calculated separately by sex, the median water intake from food among young male adults in this study (1225 mL) was close to the results of the study in 2016, and the water intake from food among young male and female adults (1173 mL) was close to the results of the study in 2018 [32]. The amount of water intake among different population groups varied widely. The fluid intake among participants in the study was lower than those of the two studies in 2016 and 2018 [9, 32]. Fluid intake and water intake from food were the two main water sources for young adults, and they accounted for a high proportion of the total water sources. This conclusion was inconsistent with the data of European and American countries. The water intake from food in European and American countries accounted for only about 20% of total water sources [1, 33]. This discrepancy may be due to the different dietary structure and habits of the population between China and European and American countries. The dietary structure of Chinese residents is mainly plant foods with rich water content, and the most common cooking methods are steaming, stewing, and frying. These cooking methods not only retain most of the original water in the food but also add water during cooking. The dietary structure of European and American countries is mainly animal food with relatively low water content, and the food cooking method is mainly baking, which loses most of the water in food [1, 33, 34]. The data on water intake from different sources would be useful to develop a recommendation on adequate water intake for the Chinese population.

This study found that the median daily urine excretion of young adults was 1295 mL. The survey among male adult college students in Hebei Province in 2016 showed that the daily urine excretion was 1358 mL [9]. Another study conducted among young adults in Hebei in 2018 showed that the daily urine excretion was 1279 mL [32]. The results of this study were similar to those of the above two studies. In this study, urine excretion accounts for 76.5% of the total water losses. Urine excretion was the main method of water loss. In this study, the ambient temperature was about 20 °C, and the relative humidity was about 50% RH. The participants did not carry out moderate- to high-intensity physical activities during the study, and they mainly carried out mild physical activities every day. Therefore, the formula used in this study was mainly to calculate the amount of non-dominant sweating, which was mainly affected by body surface area and ambient temperature and humidity. The results of this study showed that the water loss through skin evaporation and respiration accounted for 10.3% and 9.5% of the total water losses, respectively. In the study, the average number of feces was 0.7 on each of the three days. The results of the direct drying method showed that the water content of feces was about 55%. The results of water loss through feces excretion were 64 mL, and the proportion of water loss through feces excretion in the total water loss of the human body was the smallest. It was also generally recognized in some other studies that the water loss through the digestive tract by the human body is 100–300 mL every day [10, 12, 35]. The determination of water loss would be useful to estimate water requirements and develop a recommendation on adequate water intake.

This study has some strengths and weaknesses. Referring to the participants, these young adults were recruited from a university, so their sources of food and places of physical activity were similar. They were basically in an environment with relatively unified quality criteria. The age range of the participants was concentrated, which can analyze the energy expenditure, water sources, and water loss more precisely. The method used to determine the energy expenditure, sources, and loss of water was considered the gold standard. Additionally, this was the first study to analyze the amount and the proportion of water sources and losses in the human body in China. In consideration of weakness, a larger sample can provide more representative data. Only young adults, excluding people at other physiological stages, such as elderly and pregnant women, were studied. Energy value and expenditure, water sources and losses, and their proportions are affected by many factors, including age, gender, physiological stage, physical activity level, and so on. More research is needed to be conducted. In this study, plasma osmolality was also determined to reflect the hydration state of the participants. The average plasma osmolality was 290.1 mOsm/kg among 25 participants; thus, there may be a risk of dehydration. Studies with participants in optimal hydration status may provide more information and useful reference data for developing recommendations of adequate water intake.

## Conclusions

The results of this study provide reference data for the development of reference intake on energy and water intake. It is necessary to conduct more high-quality studies to determine the energy expenditure, sources, and loss of water among young adults and populations in other physiological stages or other occupations.

## Supplementary Information

**Additional file 1.** Supplementary results.

**Additional file 2.** Supplementary results of indexes related to the determination of doubly labeled water.

## Data Availability

The corresponding author will provide the data in a de-identified form used in the manuscript, code book, and analytic code available to editors upon request either before or after publication.
