# Supplementary material for: Determination of the energy expenditure, sources, and loss of water among young adults
Source: Nutr Metab (Lond). 2022 May 2;19:32. doi: 10.1186/s12986-022-00668-2 (PMC9059400; doi:10.1186/s12986-022-00668-2)
Supplement: Supplementary file 1 — Additional file 1. Supplementary results. [file 12986_2022_668_MOESM1_ESM.doc]

**Supplementary methods**

Evaluation of other influence factors

Subjective thirst sensation and urinary urgency

Subjective thirst sensation was self-evaluated using a 4-point scale: no sensation of thirst (1), initial sensation of thirst (2), strong sensation of thirst (3) and super sensation of thirst (4).

Urinary urgency was self-reported using a 4-point scale: no sensation to urinate (1), initial sensation or urge to urinate (2), strong urge to urinate(3) and super urge to urinate(4)[1].

Physical activity

Physical activity questionnaire was used to record the physical activity of the participants. The questionnaire is developed based on the international physical activity questionnaire (shortened version) and revised in accordance with expert comments. The questions included the type and time of physical activities.

Temperature, humidity and wind speed

Using the multi-functional environmental test device (MS6300; MASTECH; Shenzhen, USA), the trained investigators measured and recorded the temperature, humidity and wind speed in multi-point places including indoor and outdoor of the dormitory, classroom, canteen and playground where the participants lived. The accuracy of temperature, humidity and wind speed was 1℃, 0.1%RH and 0.1 m/s, respectively.

Statistical analysis

The formulas on the indexes related to the determination of doubly labeled water:

According to the abundance ratio of isotopes in baseline urine samples before drinking double-labeled water to the working standard (d_pre_) and the abundance ratio of isotopes in tap water at the study site to the working standard (d_tap_), the abundance ratios of ^18^O and ^2^H to the working standard measured at each time point during the study were corrected and converted by the following formula:

^18^O/^2^H abundance=(d_post -_ d_pre_)/ (d_dose -_ d_tap_) × (18.02a/WA); d_dose_, means the abundance ratio of isotopes after dilution to the working standard; a,W,A, means the weight of double-labeled water for analysis, the weight of all distilled water used for diluting double-labeled water and the weight of doubly labeled water drunk by participants[2].

Taking the natural logarithm of the corrected ^18^O and ^2^H abundance ratio in each time point as the ordinate and the test time as the abscissa, two isotope elimination curves was drawn. According to the correlation coefficient of the curve fitting formula, the ^18^O and ^2^H rate constant (K_O_ and K_D_) was obtained. The reciprocal of the anti-logarithm intercepted by the curve was the dilution space of the two isotopes (N_O_ and N_D_). Referring to the Speakman model, the generation rate of CO_2_ was calculated using the following formulas[3]:

*rCO_2_=(N/2.196) ×(K_O_ -1.0427 K_D_); N=( N_O_ + N_D_/1.0427)/2*

According to the analysis that the participants were in a constant state of weight during the study, the food quotient (FQ) was used to replace the respiratory quotient (RQ), and the FQ was obtained by calculating the proportion of the three energy supplying substances in the intake of food[4,5]. The consumption rate of O_2_ and TEE were calculated using the following formulas[6]:

*VO_2_ (L/g)=0.966×protein (g)+2.019×fat (g)+0.829×carbohydrate (g); VCO_2_, means carbon dioxide production*；*VO_2_, means oxygen consumption*

*VCO_2_ (L/g)==0.774×protein (g)+1.427×fat (g)+0.829×carbohydrate (g)；VCO_2_, means carbon dioxide production*

*TEE (kJ/d)=(3.9×VO_2_+1.1×VCO_2_) ×4.184*

*FQ= VCO_2_/ VO_2_*

*rO_2_= rCO_2_/FQ*

*The calculation on the metabolic water, water loss through skin evaporation and respiration:*

The metabolic water, water loss through skin evaporation and respiration were calculated using the following formulas[7,8]:

*The metabolic water (L/d)=TEE (kcal/d) ×(1/10^5^) ×[(%fat×0.119)+ (%protein×0.103)+ (%carbohydrate×0.15)+ (%alcohol×0.168)]*

*Water loss through skin evaporation (L/d)=[0.18×absolute humidity (mg/L)/21.7×body surface area (m^2^)×1.44; absolute humidity was converted from measured relative humidity and temperature values; body surface area(m^2^)=height^0.725^ (m) ×weight^0.425^ (kg) ×0.007184*

*Water loss through respiration (L/d)=Respiratory volume (L/d) ×absolute humidity(mg/L)/1000*

*Reference*

1. Athwal; B., S. Brain responses to changes in bladder volume and urge to void in healthy men. *Brain* **2001**, *124*, 369-377.

2. Liu, J.; Yang, X.; Piao, J.; Sun, R.; Tian, Y.; Tian, Y. The Energy Expenditure Determined by the Doubly Labeled Water Method in 16 Young Adult Women. *Acta Nutrimenta Sinica* **2010**, *32*, 216-220.

3. Speakman, J.R.; Nair, K.S.; Goran, M.I. Revised equations for calculating CO2 production from doubly labeled water in humans. *American Journal of Physiology* **1993**, *264*, 912-917.

4. Goris, A.; Westerterp, K.R. Postabsorptive respiratory quotient and food quotient-an analysis in lean and obese men and women. *European Journal of Clinical Nutrition* **2000**, *54*, 546-550.

5. Westerterp, K.R. Food quotient, respiratory quotient, and energy balance. *American Journal of Clinical Nutrition* **1993**, *57*, 759S-764S.

6. Weir; Bdv, J. New methods for calculating metabolic rate with special reference to protein metabolism. *J. Physiol* **1949**, *109*, 1-9.

7. Sagayama, H.; Kondo, E.; Shiose, K.; Yamada, Y.; Motonaga, K.; Ouchi, S.; Kamei, A.; Osawa, T.; Nakajima, K.; Takahashi, H. Energy Requirement Assessment and Water Turnover in Japanese College Wrestlers Using the Doubly Labeled Water Method. *Journal of Nutritional Science and Vitaminology* **2017**, *63*, 141-147.

8. Bois, D.; Bois, E. A Formula to Estimate the Approximate Surface Area if Height and Weight be Known. *Nutrition* **1989**, *5*, 303-311.
