# Supplementary material for: Determination of the energy expenditure, sources, and loss of water among young adults
Source: Nutr Metab (Lond). 2022 May 2;19:32. doi: 10.1186/s12986-022-00668-2 (PMC9059400; doi:10.1186/s12986-022-00668-2)
Supplement: Supplementary file 2 — Additional file 2. Supplementary results of indexes related to the determination of doubly labeled water. [file 12986_2022_668_MOESM2_ESM.doc]

Supplementary results

Indexes related to the determination of doubly labeled water

Indexes related to the determination of doubly labeled water was shown in the supplementary file.

The δ values of ^2^H and ^18^O in urine samples were converted into atom percent excess (APE) according to the formula, respectively. After taking the natural logarithm of APE as the ordinate and time (T) as the abscissa, two isotope elimination curves were drawn. The ^2^H isotope elimination curve in urine sample was *y=-0.1213x-7.4447, R^2^*=0.9916. And the ^18^O isotope elimination curve in urine sample was *y=-0.1503x-7.4552, R^2^*=0.9942(Supplementary Figure 1 and 2).


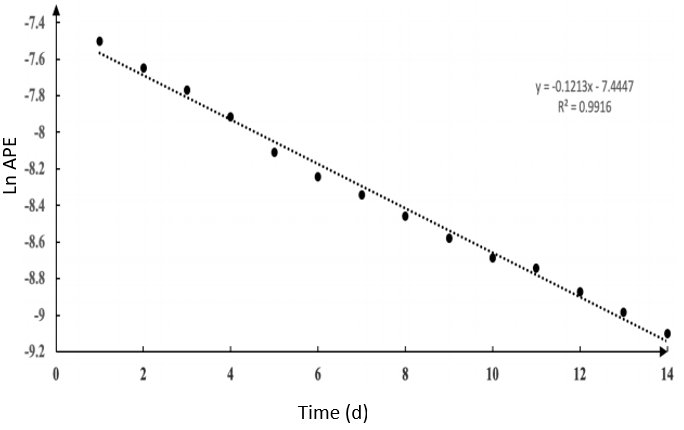


**Supplementary Figure 1.** The ^2^H isotope elimination curve in urine sample.


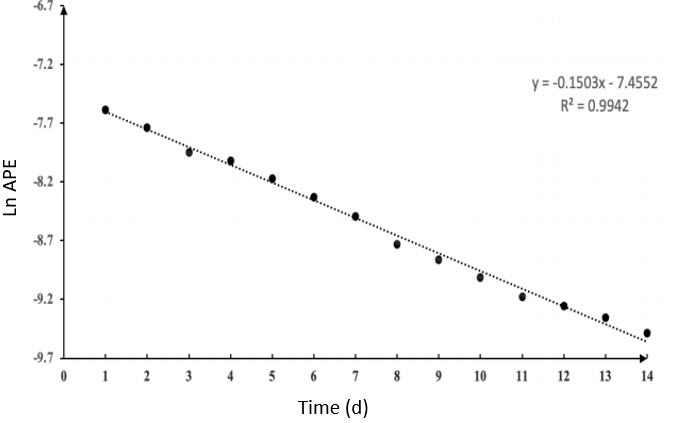


**Supplementary Figure 2.** The ^18^O isotope elimination curve in urine sample.

By checking the enrichment of ^2^H and ^18^O in urine samples collected on day 14, the ^18^O abundances of all 25 participants were more than 8‰ and the ^2^H abundances of all 25 participants were more than 128‰, which could avoid the random error in the measurement of isotopic abundances. By comparing the dilution space of ^18^O and ^2^H, the ratio of N_O_/N_D_ was 1.011, and the ratio was between 1.000-1.070. The ideal ratio for adults is 1.034. The ratio of isotope elimination rate (K_O_:K_D_) was 1.239, and the ratio was between 1.1 and 1.7. The above values met the requirements on quality control indexex recommended in the isotope application guide issued by the International Atomic Energy Agency (IAEA)[30]. According to the formulas, the FQ value of female and male was 0.882±0.003 and 0.874±0.004, respectively. And the rCO_2_ of female and male was 14.87±0.94 and 14.73±0.94, respectively (Supplementary Table 1).

**Supplementary Table 1.** Indexes related to the determination of doubly labeled water.

|  | **Female** | **Male** |
| --- | --- | --- |
| Rate constant (d^-1^) |  |  |
| K_O_ | 0.1244±0.01 | 0.1308±0.01 |
| K_D_ | 0.1085±0.02 | 0.1101±0.02 |
| Dilution space(mol) |  |  |
| N_O_ | 1631.32±95.02 | 1725.52±94.48 |
| N_D_ | 1614.64±96.00 | 1709.35±94.49 |
| N_O_/N_D_ | 1.01±0.01 | 1.01±0.01 |
| FQ | 0.874±0.004 | 0.882±0.003 |
| rCO_2_(mol/d) | 14.73±0.94 | 14.87±0.94 |

Note: Values were shown as the mean±standard deviation (SD); K_O_ and K_D_ was the turnover rate of ^2^H and ^18^O , respectively; N_O_ and N_D_ was the dilution space of ^2^H and ^18^O, respectively; FQ was the food quotient obtained by calculating the proportion of the three energy supplying substances in the intake of food; rCO_2_ was the generation rate of CO_2_.

1. Athwal; B., S. Brain responses to changes in bladder volume and urge to void in healthy men. *Brain* **2001**, *124*, 369-377.

2. Liu, J.; Yang, X.; Piao, J.; Sun, R.; Tian, Y.; Tian, Y. The Energy Expenditure Determined by the Doubly Labeled Water Method in 16 Young Adult Women. *Acta Nutrimenta Sinica* **2010**, *32*, 216-220.

3. Speakman, J.R.; Nair, K.S.; Goran, M.I. Revised equations for calculating CO2 production from doubly labeled water in humans. *American Journal of Physiology* **1993**, *264*, 912-917.

4. Goris, A.; Westerterp, K.R. Postabsorptive respiratory quotient and food quotient-an analysis in lean and obese men and women. *European Journal of Clinical Nutrition* **2000**, *54*, 546-550.

5. Westerterp, K.R. Food quotient, respiratory quotient, and energy balance. *American Journal of Clinical Nutrition* **1993**, *57*, 759S-764S.

6. Weir; Bdv, J. New methods for calculating metabolic rate with special reference to protein metabolism. *J. Physiol* **1949**, *109*, 1-9.

7. Sagayama, H.; Kondo, E.; Shiose, K.; Yamada, Y.; Motonaga, K.; Ouchi, S.; Kamei, A.; Osawa, T.; Nakajima, K.; Takahashi, H. Energy Requirement Assessment and Water Turnover in Japanese College Wrestlers Using the Doubly Labeled Water Method. *Journal of Nutritional Science and Vitaminology* **2017**, *63*, 141-147.

8. Bois, D.; Bois, E. A Formula to Estimate the Approximate Surface Area if Height and Weight be Known. *Nutrition* **1989**, *5*, 303-311.
